# EFEMP1 is a potential biomarker of choroid thickness change in myopia

**DOI:** 10.3389/fnins.2023.1144421

**Published:** 2023-02-20

**Authors:** Wen-Qing Shi, Ting Wan, Bing Li, Tao Li, Xiao-Dong Zhou

**Affiliations:** ^1^Department of Ophthalmology, Jinshan Hospital of Fudan University, Shanghai, China; ^2^Central Laboratory, Jinshan Hospital of Fudan University, Shanghai, China

**Keywords:** myopia, choroid thickness, EFEMP1, OCT, FDM

## Abstract

**Purpose:**

To explore the possible molecular mechanism by which epidermal growth factor-containing fibulin-like extracellular matrix protein 1 (EFEMP1) regulates choroid thickness (CT) in the development of myopia.

**Methods:**

In total, 131 subjects were divided into the emmetropia (EM) group, non-high myopia (non-HM) group and high myopia (HM) group. Their age, refraction, intraocular pressure, and other ocular biometric parameters were collected. A 6 × 6 mm area centered on the optic disc was scanned by coherent optical tomography angiography (OCTA) to measure CT, and the tear concentrations of EFEMP1 were quantified using enzyme-linked immunosorbent assay (ELISA) analysis. Twenty-two guinea pigs were divided into the control group and the form-deprivation myopia (FDM) group. The right eye of the guinea pig in the FDM group was covered for 4 weeks, and the diopter and axial length of the right eye of the guinea pig were measured before and after the treatment. After the measurement, the guinea pig was euthanized, and the eyeball was removed. Quantitative reverse transcription polymerase chain reaction, western blotting assays and immunohistochemistry were used to assess the expression of EFEMP1 in the choroid.

**Results:**

There were significant differences in CT among the three groups (*p* < 0.001). CT was positively correlated with age in HM (*r* = −0.3613, *p* = 0.0021), but no significant correlation with SE (*p* > 0.05) was observed. Furthermore, there were increased levels of EFEMP1 in the tears of myopic patients. After 4 weeks of covering the right eye of the FDM guinea pigs, there was a significant increase in axial length and a decrease in diopter (*p* < 0.05). The mRNA and protein expression of EFEMP1 was significantly increased in the choroid.

**Conclusion:**

Choroidal thickness was significantly thinner in myopic patients, and the expression level of EFEMP1 in the choroid increased during the development of FDM. Therefore, EFEMP1 may be involved in the regulation of choroidal thickness in myopia patients.

## Introduction

Myopia has become a growing global threat to public health in recent years (Baird et al., 2020). In Asia, the prevalence of myopia is over 80%. Myopia is a major risk factor for ocular diseases, such as cataracts, glaucoma, and choroidal neovascularization, and it is also associated with similar levels of risk for hypertension, coronary heart disease, and stroke (Baumgarten et al., 2018). According to a study by Naidoo et al. (2019), the potential global economic loss associated with myopia in 2015 was nearly $250 billion. Hence, myopia is a medical problem as well as a heavy social problem that has received increasing attention.

Studies have shown that the choroid plays an important role in regulating ocular development. Blood vessels are a major component of the choroid, and studies have shown that choroidal vessel density decreases with age in healthy people over 30 years old (Fujiwara et al., 2016), while changes in choroidal vessel lumen area can directly affect choroidal thickness (Li et al., 2018). Changes in choroidal thickness (CT) affect the diffusion dynamics between the retina and the sclera, mediating the effect of retinal signals on the sclera and thus regulating scleral growth and refractive state of the eye (Summers, 2013). Numerous previous studies have found that the choroid thickness in myopia differs significantly from emmetropia (Tian et al., 2021; Xiuyan et al., 2021). Nishida et al. (2012) found a negative correlation between CT and age and diopter in patients with high myopia, which is an important predictor of visual acuity. Similarly, consistent findings were obtained in animal models of myopia, where CT was found to be significantly thinner in both chicks and guinea pigs after myopia modeling (Fitzgerald et al., 2002; Lu et al., 2009; Zhang et al., 2019). However, the mechanism leading to CT changes is not clear.

Epidermal growth factor-containing fibulin-like extracellular matrix protein 1 (EFEMP1) is a 55 kDa disulfide-bonded secreted extracellular matrix glycoprotein widely expressed in epithelial and endothelial cells (Cheng et al., 2020). Numerous studies have found that EFEMP1 is highly expressed in elastin-rich tissues of human and mouse eyes, especially in the corneal and choroidal retinal pigment epithelium (Livingstone et al., 2020). EFEMP1 is typically characterized by a structural domain rich in epidermal growth factor (EGF), while EGF has been proven to be highly associated with myopia (Jonas et al., 2021). Meanwhile, Daniel et al. (2020) observed structural deformities in the cornea by altering the expression of EFEMP1. The cornea is part of the refractive system. These studies suggest that EFEMP1 may play a role in the development of myopia. In addition, Cheng et al. (2020) found that EFEMP1-overexpressing HUVECs showed a significant increase in tube formation and proliferation. Since CT depends on its perfusion rate, we speculate that EFEMP1 may act as a signaling molecule to regulate the alteration of CT in the development of myopia.

To date, whether the protein levels of EFEMP1 in the choroid change during the development of form-deprivation myopia (FDM) has not been reported. Therefore, we scanned the choroid of myopic subjects using OCTA to analyze the changes in thickness, and EFEMP1 levels in the participants’ tears were measured and subsequently validated in the choroidal tissue of FDM guinea pigs to investigate the possible molecular mechanisms involved in the regulation of CT changes by EFEMP1 in myopic patients.

## Materials and methods

### Study subjects

One hundred thirty-one subjects (131 eyes) were recruited at Jinshan Hospital of Fudan University from October 2019 to October 2021. The inclusion criteria of all subjects were: (1) age range from 18 to 70 years old, (2) spherical equivalence (SE) ≤ + 0.5 D, (3) intraocular pressure ≤21 mmHg, (4) no ocular lesions, and (5) right-handedness. The exclusion criteria were: (1) smokers and alcoholics, (2) eye diseases, (3) systemic diseases, such as diabetes and hypertension, (4) pregnant or breastfeeding patients, (5) long-term chronic treatment, and (6) history of ophthalmic surgery. All subjects were divided into three groups: (1) Emmetropia (EM) group: SE ranged from −0.5 to +0.5 (D); (2) Non-high myopia (Non-HM) group: SE > −6.0 D; (3) High myopia (HM) group: SE ≤ −6.0 D. All examinations were performed with the patient’s informed consent.

### Clinical examinations

All adult subjects underwent ocular examination, including diopter (CT-5000; Topcan Corporation, Tokyo, Japan), best corrected visual acuity (BCVA) in angle of minimum resolution (LogMAR), intraocular pressure measurement (CT- 80; Canon Inc., Tokyo, Japan), slit lamp examination (SL-F7; Topcan Corporation, Tokyo, Japan), and fundus photography (VISUCAM 200; Carl Zeiss, Jena, Germany). Eyeball biological parameters were measured in 131 adult subjects using A-ultrasound (Aviso; Quantel, France) and IOL-Master 500 (Carl Zeiss, Jena, Germany), respectively.

### Image acquisition and analysis

**FIGURE 1:**
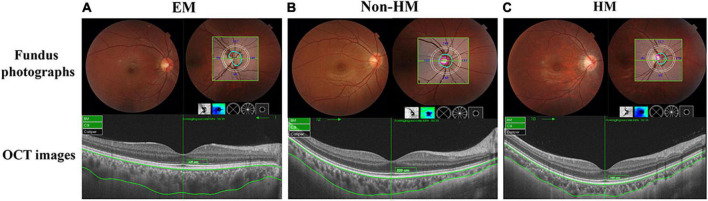
Representative fundus photographs, optical coherence tomography (OCT) images, and thickness graph of EM **(A)**, non-HM **(B)**, and HM **(C)** groups. EM, emmetropia; HM, high myopia.

Fundus scans of the optic disc area were performed on all subjects using a 6 mm × 6 mm 3D Disc mode in Triton OCTA (Topcan, Tokyo, Japan) (Liu et al., 2020). The choroid was automatically stratified using OCTA software, and the measurement partitions were divided into four quadrants (S, Superior; I, Inperior; N, Nasal; T, Temporal) according to the diagonal of the two quadrants by the Early Treatment Diabetic Retinopathy Study (ETDRS) (Figure 1). The exclusion criteria for OCTA examination were signal intensity index <40 and images with severe artifacts due to poor eye fixation.

### Tear collection and preservation

A 0.5 mm diameter polyethylene capillary was placed in the conjunctival sac of the lower dome, and 5–20 μL of tears were collected from both eyes by siphoning into Eppendorf tubes and stored at −80°C for examination.

### Enzyme-linked immunosorbent assay

A human EFEMP1 ELISA kit (ZN2177; Xi’an Baiolaib Biotechnology Co., Ltd) was used for the determination of EFEMP1 content in tear fluid. One hundred microliters of tear fluid per tube was used for enzyme-linked immunosorbent assay (ELISA) analysis. EFEMP1 content in tear fluid was measured after preparation of 3.125–100 ng/ml standards (Supplementary Figure 1).

### Animal experiments

Two-week-old, 100–150 g tricolor male guinea pigs were purchased from Danyang Changyi Laboratory Animal Breeding Co (Jiangsu, China). All animals are kept in natural light conditions to ensure an adequate daily supply of food, water, and fresh vegetables. All animal experiments were approved by Shanghai Public Health Clinical Center Laboratory Animal Welfare & Ethics Committee and in accordance with the ARVO Statement on the Use of Animals in Ophthalmic and Vision Research.

### Animal grouping and model preparation

Twenty-two guinea pigs were randomly divided into the FDM group and the control group. For the FDM group, a translucent latex balloon mask was used to cover the right eye, and all other parts of the face were exposed. No treatment was used for the control group. All animals were kept in a natural light environment.

### Measurement of ocular biometric features

All guinea pigs were labeled and numbered, and SE was measured in a dark room with streak retinoscopy (YZ24; Six-Six Vision Technology Co., Ltd). Measurements were averaged three times and were accurate to 0.01 D. The conjunctival sac was filled with 1% tropicamide drops three times, each time at 5 min intervals, and the axial length of the eye was measured using A-scan ultrasound (OD1-A, Kaixin Electronic Instrument Co., Ltd., China). Manual measurements were averaged 10 times and were accurate to 0.01 mm. Fundus photography was used to take pictures of the fundus of the right eye of each guinea pig after anesthesia (VISUCAM 200; Carl Zeiss, Jena, Germany).

### Tissue preparation

The animals were euthanized by overdose with an intraperitoneal injection of sodium pentobarbital. The right eyeball was removed, and a portion of the eyeball was separated from the choroidal tissue, quickly placed in a lyophilization tube and stored at −80°C for western blot experiments. The remaining eyeball was fixed by perfusion and paraffin-embedded for immunohistochemical experiments.

### Quantitative reverse transcription polymerase chain reaction

Extraction of total RNA from choroidal tissues using Tissue RNA Purification Kit (Yishan Biotechnology, China). The gene transcription was quantified by quantitative RT-PCR with PrimeScript™ RT Master Mix Kit (Takara, Shiga, Japan). The sequence of the primers are shown in Table 1 (Sangon Biotechnology, China).

**TABLE 1:** The primer sequences.

Primer	Forward 5′-3′	Reverse 5′-3′
EFEMP1	GGACGCACAACTGTAGAGCAG AC	CTTTGGTGGCAATATGGAGGG ATGG
β-actin	CTGGGTATGGAATCCTGTGGC ATC	CTGTGTTGGCATAGAGGTCCT TACG

### Western blotting

Frozen choroidal tissues were added proportionally (10 mg) to 100 μL of radioimmunoprecipitation assay buffer (Beyotime, China), and 1 mm phenylmethanesulfonyl fluoride (PMSF, Beyotime, China) was mixed and then homogenized on ice using a tissue homogenizer. Protein concentrations were then determined using a BCA protein assay kit (Beyotime, China). Protein samples (25 μg) were separated by 10% SDS–PAGE and transferred to a polyvinylidene difluoride (PVDF) membrane. After blocking with 5% skim milk for 1 h at room temperature, the membrane was incubated with EFEMP1 antibody (1:1000, ab106429, Abcam, Cambridge, MA, USA) at 4°C overnight and then incubated with species-specific HRP-conjugated secondary antibodies (diluted 1:5000, CoWin Biosciences, Cambridge, MA, USA) for 1 h at room temperature. Then, radioautography-enhanced chemiluminescence (ECL, Thermo Scientific) was performed. β-actin (1:5000, 69,009-1, ProteinTech, Chicago, IL, USA) was used as an internal standard. The grayscale values of each band were calculated and statistically analyzed.

### Immunohistochemistry

Paraffin-embedded sections containing eyeball tissue were deparaffinized in a 60°C oven for 50 min and then rehydrated in xylene and graded alcohol solutions. First, tissue antigens were extracted in a rice cooker at 95°C containing citrate antigen retrieval solution, and tissue sections were then incubated in 3% H_2_O_2_ solution for 15 min at room temperature to inactivate endogenous peroxidase. Nonspecific binding was blocked with goat serum for 10 min at room temperature. Slides were incubated overnight at 4°C with EFEMP1 primary antibody (ab106429, Abcam, Cambridge, MA, USA) diluted to 1:50. After 18 h, the slides were washed with PBS (0.01 M; pH 7.4) for 5 min, which was repeated three times. After incubating with secondary antibody for 30 min, a 3,3′-diaminobenzidine-dine chromogen kit was used for staining, followed by staining with hematoxylin for 2.5 min at room temperature, rinsing the sample with tap water, using dilute hydrochloric acid to acidify the hematoxylin and allowing the slide to dry. Then, they were sealed with neutral gum. PBS was used as a negative control instead of primary antibody.

### Statistical analysis

All data were analyzed using the statistical software SPSS (version 25, SPSS Inc., Chicago, IL, USA). Data are presented as the means ± SEM. Comparisons between groups were performed using independent *t* test, one-way ANOVA, Welch, and Brown-Forsythe test followed by Bonferroni and Tamhini multiple comparison tests. A *p*-value < 0.05 was considered statistically significant.

## Results

### General information and ocular parameters

**FIGURE 2:**
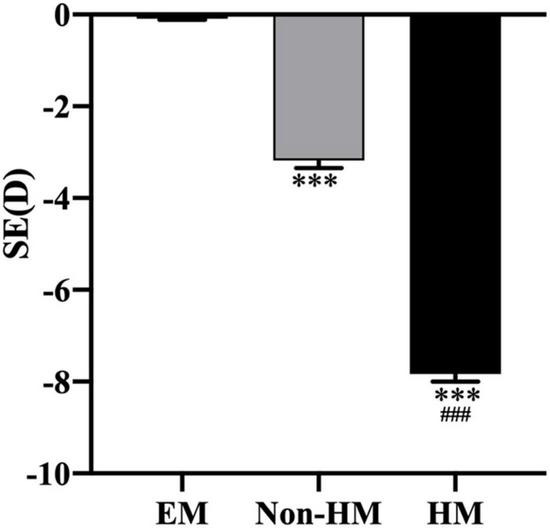
Spherical equivalent in the right eyes of the three groups. ^***^p < 0.001, compared with EM group; ^###^*p* < 0.001, compared with non-HM group. SE, spherical equivalent; EM, emmetropia; HM, high myopia.

**FIGURE 3:**
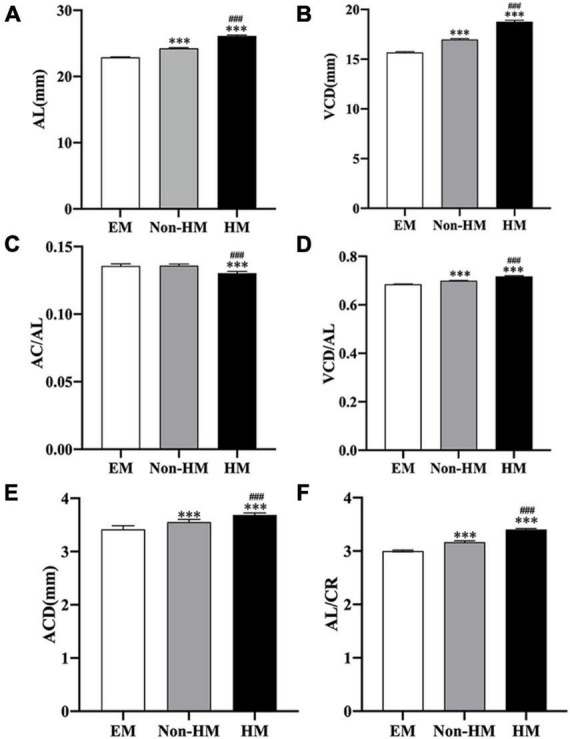
Ocular biometric parameters of the right eye measured by A-scan and IOL-Master. **(A)** AL in three groups. **(B)** VCD in three groups. **(C)** AC/AL in three groups. **(D)** VCD/AL in three groups. **(E)** ACD in three groups. **(F)** AL/CR in three groups. ^***^p < 0.001, compared with EM group; ^###^*p* < 0.001, compared with non-HM group. EM, emmetropia; HM, high myopia; AL, axial length; VCD, vitreous chamber depth; AC, anterior chamber depth; ACD, anterior chamber depth; CR, radius of corneal curvature.

In total, 131 subjects (51 females and 80 males) were collected in this study and divided into EM (−0.5 D ≤ SE ≤+ 0.5 D), non-HM (−6 D < SE < −0.5 D), and HM groups (SE ≤ −6 D); the mean SE of the three groups were −0.15 ± 0.25 D, −3.31 ± 1.46 D, and −7.70 ± 1.36 D, respectively. There was a significant difference in SE among the three groups (*p* < 0.001) (Figure 2 and Table 2). The demographic characteristics and clinical data of the subjects in the three groups are shown in Figures 3 and Supplementary Tables 1, 2.

**TABLE 2:** Demographics and clinical measurements by groups.

Parameter	EM	Non-HM	HM	*p*-value	*p*-value_1_	*p*-value_2_	*p*-value_3_
Sample Size	15	46	70	NA	NA	NA	NA
Age(years)	33.67 ± 12.12	36.39 ± 12.29	33.90 ± 12.09	0.721	1.000	1.000	1.000
SEX, F/M	7/8	16/30	28/42	0.698	–	–	–
SE(Diopter)	−0.15 ± 0.25	−3.31 ± 1.46	−7.70 ± 1.36	<0.001	<0.001	<0.001	<0.001
BCVA, logMAR	−0.01 ± 0.05	0.00 ± 0.02	0.05 ± 0.12	0.002	0.573	0.002	0.002
IOP(mmHg)	15.40 ± 2.53	14.96 ± 1.71	14.83 ± 2.63	0.737	0.899	0.824	0.985

*p*-value among the three groups; *p*-value_1_, *p*-value EM and non-HM; *p*-value_2_, *p*-value between EM and HM; *p*-value_3_, *p*-value between non-HM and HM. EM, emmetropia; HM, high myopia; NA, not applicable; SE, spherical equivalent.

### CT in different choroid regions and correlation between CT, SE, and age

**FIGURE 4:**
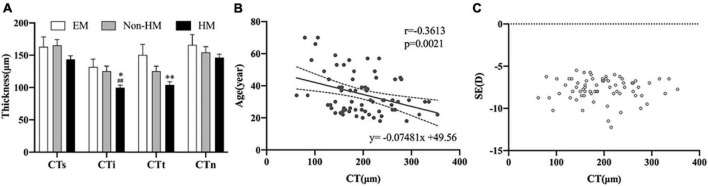
Choroidal thickness of right eye. **(A)** Thickness in three groups. **(B)** Scatter plot of correlation between age and CT. **(C)** Scatter plot of correlation between SE and CT. ^**^p < 0.01, compared with EM group; ^##^*p* < 0.01, compared with non-HM group. EM, emmetropia; HM, high myopia; CT, choroid thickness; SE, spherical equivalence; s, superior; i, inferior; t, temporal; n, nasal. *p < 0.05.

The mean choroidal thicknesses of the EM group, non-HM group, and HM group were 270.87 ± 62.01 μm, 233.76 ± 80.94 μm, and 195.91 ± 63.23 μm, respectively, with significant differences between the three groups (*p* < 0.001). The segmentation of choroidal regions using ETDRS showed that the thickness of CTi and CTt in the EM group was significantly thinner than that in the HM group (*p* < 0.05), and the thickness of CTi in the HM group was significantly thinner than that in the non-HM group (*p* < 0.01) (Table 3). In the high myopia group, there was a significant positive correlation between CT and age (*r* = −0.3613, *p* = 0.0021), while there was no significant correlation with SE (*p* > 0.05) (Figure 4). Signal intensity index of OCTA examination was >40 in all subjects.

**TABLE 3:** Choroidal parameters in the participant’s right eye.

Parameter	EM	Non-HM	HM	*p*-value	*p*-value_1_	*p*-value_2_	*p*-value_3_
Sample size	15	46	70	NA	NA	NA	NA
CT (μm)	270.87 ± 62.01	233.76 ± 80.94	195.91 ± 63.23	<0.001	0.029	0.001	0.015
CTs (μm)	163.00 ± 58.96	165.33 ± 60.58	143.57 ± 46.52	0.079	1.000	0.606	0.100
CTi (μm)	131.87 ± 47.00	125.43 ± 52.86	99.67 ± 34.91	0.005	1.000	0.03	0.006
CTt (μm)	150.33 ± 63.65	125.35 ± 52.90	104.19 ± 40.33	0.009	0.247	0.003	0.065
CTn (μm)	165.87 ± 62.35	154.50 ± 59.18	146.40 ± 45.19	0.383	0.903	0.607	0.817

*p*-value among the three groups; *p*-value_1_, *p*-value EM and non-HM; *p*-value_2_, *p*-value between EM and HM; *p*-value_3_, *p*-value between non-HM and HM. EM, emmetropia; HM, high myopia; NA, not applicable; CT, choroid thickness; s, superior; i, inferior; t, temporal; n, nasal.

### Tear concentrations of EFEMP1 and correlations between EFEMP1 levels and ocular biometric parameters

**FIGURE 5:**
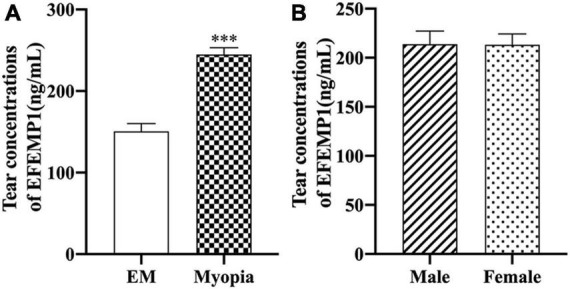
EFEMP1 concentrations of tear between different groups. **(A)** Tear EFEMP1 concentration in emmetropia group and myopic group. **(B)** EFEMP1 concentration of tears in male and female groups. ^***^p < 0.001, compared with EM group. EM, emmetropia.

**FIGURE 6:**
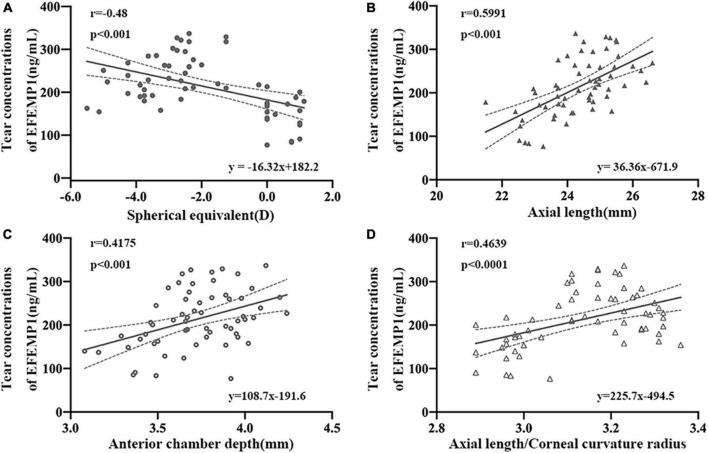
Correlation between tear concentrations of EFEMP1 and ocular biometric parameters in two groups. **(A)** Spherical equivalence. **(B)** Axial length. **(C)** Anterior chamber depth. **(D)** Axial length/Corneal curvature radius. •, ∘, △, ▲: scatter plot.

The average levels of EFEMP1 tears in the myopia group and the emmetropia group were 150.53 ± 42.37 ng/ml and 244.89 ± 51.35 ng/ml, respectively, with a significant difference between the two groups (*p* < 0.001) (Figure 5). Tear concentrations of EFEMP1 in the myopia group were negatively correlated with SE (*r* = −0.4800, *p* < 0.001), and AL (*r* = 0.5991, *p* < 0.001), ACD (*r* = 0.4175, *p* < 0.001), and AL/CR (*r* = 0.4639, *p* < 0.001) were positively correlated (Figure 6).

### Diopter and axial length and expression of EFEMP1 mRNA and protein in guinea pigs

**FIGURE 7:**
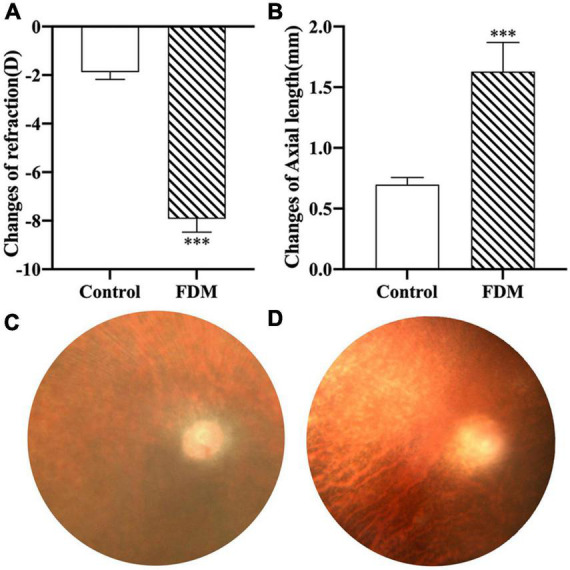
Changes in right eye diopter and axial length in two groups. **(A)** Changes of refraction. **(B)** Changes of axial length. **(C)** Fundus of guinea pigs in control group. **(D)** Fundus of guinea pigs in FDM group. ^***^p < 0.001, compared with control group. FDM, form-deprivation myopia.

**FIGURE 8:**
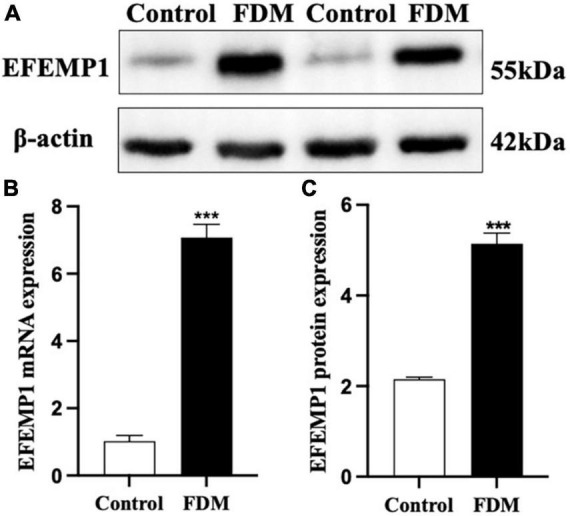
Expression of EFEMP1 mRNA and protein. **(A)** Western blot image of the two groups. **(B)** mRNA expression of EFEMP1. **(C)** Protein expression of EFEMP1. ***p < 0.001, compared with control group, *n* = 5.

After 4 weeks of covering, the FDM group showed a significant decrease in diopter (*p* < 0.001), a gradual increase in axial length (Figure 7), and a significant increase in EFEMP1 mRNA and protein expression in the choroid compared with the control group (all *p* < 0.001) (Figure 8).

### EFEMP1 immunohistochemical staining

**FIGURE 9:**
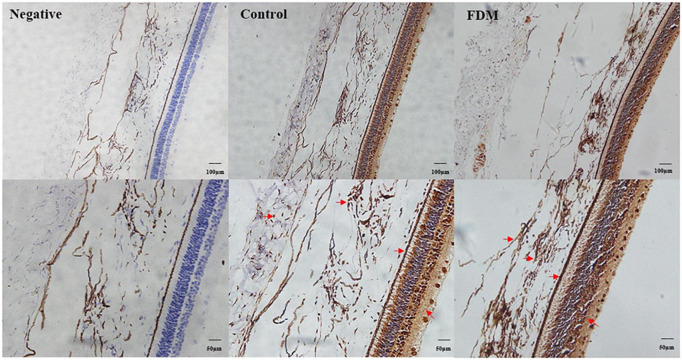
Immunohistochemical staining of EFEMP1. A brown stain (red arrow) represents the location of EFEMP1 and a blue stain represents the nucleus.

As shown in Figure 9, EFEMP1 was mainly localized in the ganglion cell layer, inner plexiform layer, inner nuclear layer, RPE layer, scleral extracellular matrix, choroidal vessel wall, and other structures.

## Discussion

The choroid is a highly vascularized tissue structure located between the retina and the sclera. In addition to providing oxygen and nutrients to the outer retina, it also mediates visual signals to regulate refractive development and plays an important role in the process of emmetropia or myopia progression (Nickla and Wallman, 2010). Histological studies have suggested that choroidal thickness is closely related to changes in choroidal blood flow (CBF) (Fitzgerald et al., 2002), and numerous studies have been conducted to measure changes in CBF between myopia and emmetropia groups, yet the results have been inconsistent. Some studies have concluded insignificant changes in CBF between myopia and emmetropia (Milani et al., 2018), but others have shown a significant decrease in CBF in myopic patients (Wu et al., 2021). Furthermore, Al-Sheikh et al. (2017) found that blood flow density in the retinal layer was reduced in highly myopic eyes compared to controls, while blood perfusion in the choroidal capillary layer was increased. The inconsistency of the results of the above studies may be related to various factors, such as the observation site and the sensitivity of the instrument. As an objective indicator of choroidal changes, CT provides a clearer indication of the role it may play in myopia development than CBF (Ohno-Matsui and Jonas, 2019). An abundance of studies has found that CT decreases with increasing axial length, especially in patients with high myopia (Duan et al., 2019). In the present study, we found significant differences in CT values among the three groups of subjects, in particular, the HM group was significantly different from the other two groups, suggesting a possible association between the myopia degrees and choroidal thicknesses, which is consistent with the results of previous studies (Deng et al., 2018).

In the process of searching for the molecular mechanism of choroid changes in myopia patients, the collection of tears from myopia patients is a feasible operation, which has the advantages of being noninvasive and acceptable. At present, tear detection is mainly used for the analysis of ocular surface diseases, such as dry eye and conjunctivitis (O’Neil et al., 2019). Fewer studies have observed changes in tear composition in myopia studies; however, we consider this to be an exploration of ways to find relevant molecular mechanisms. First, tears are secreted by lacrimal glands and conjunctival goblet cells. The blood flow of the lacrimal gland, conjunctiva, and choroid all belong to the branches of ophthalmic arteries and return to the cavernous sinus through the superior ophthalmic vein (Wang, 2002), with a certain homology between the three, which may indirectly reflect molecular changes in the choroid or in the eye. Second, tears have been used as a biomarker to assess the progression and prognosis of ophthalmic diseases, such as cataracts and age-related macular degeneration (Brown et al., 2018; Engelbrecht et al., 2020). This study is the first to report the relationship between the tear concentration of EFEMP1 and biological parameters of myopic eyes. We found that the EFEMP1 concentration in myopic tears was significantly increased and showed a significant negative correlation with SE and a significant positive correlation with AL, ACD, and AL/CR, suggesting that upregulation of EFEMP1 may be associated with the development of axial myopia.

Combined with the decrease in choroid thickness in myopic patients and the significant increase in EFEMP1 concentrations in the tears of myopic patients in this study, we speculated that EFEMP1 might be involved in the molecular mechanism of CT changes during the development of myopia. Due to the limitations of tear detection, we subsequently verified this hypothesis with FDM guinea pigs and found that EFEMP1 was significantly increased in choroid tissue. However, how EFEMP1 acts on downstream molecules and the possible specific mechanism of EFEMP1 in the FDM process need to be further studied.

Previous studies have found that altered EFEMP1 expression is closely associated with many ocular diseases (Springelkamp et al., 2015), mutations in EFEMP1 expression (R345 W) may lead to Malattia Leventinese (Tsai et al., 2021), and the expression of EFEMP1 is abnormally high in the ciliary body of patients with open-angle glaucoma (Collantes et al., 2022). However, studies on changes in EFEMP1 protein in the choroid in myopia models have not been reported. EFEMP1 contains 493 amino acids and belongs to a family of proteins containing the EGF structural domain. All known sequences of epidermal growth factor receptor (EGFR)-binding proteins have been compared with EFEMP1 sequences, and it was found that EFEMP1 has high homology with EGF and interacts with EGFR (Camaj et al., 2009; Chu and Peters, 2008). Dong et al. (2020) showed that EGFR expression is significantly increased in myopic guinea pigs and that intravitreal injection of EGFR antibody is dose-dependently associated with axial elongation of lens-induced myopia in guinea pigs, suggesting that EFEMP1 may synergize with EGFR in myopia regulation. In addition, (Roybal et al., 2005) found that overexpression of EFEMP1 in RPE cells effectively activated the unfolded protein response (UPR), leading to upregulation of vascular epidermal growth factor (VEGF) expression. UPR is thought to be a protective mechanism for cells against external stimuli, but when endoplasmic reticulum stress is persistent, UPR is unable to correct the imbalance protein homeostasis and initiates apoptosis-related signaling. Studies have shown that as myopia progresses, especially high myopia, RPE cell density decreases (Zhang and Wildsoet, 2015). Meanwhile, anti-VEGF was proven to have an inhibitory effect on myopia development in a chick model of deprivation myopia (Mathis et al., 2014). Scholars have found that vascular endothelial factor A (VEGF-A) is highly expressed in the choroidal vessel wall and may play a large role in the response to choroidal thickening caused by myopic defocusing (Mathis et al., 2014). Enhanced EFEMP1 expression around the choroidal canal wall was seen in the IHC results of this study. Therefore, we speculate that the increase in EFEMP1 may be associated with the increase in EGFR and VEGF during myopia development. Moreover, EFEMP1 is important for maintaining the integrity of the basement membrane and the binding of other extracellular matrices, such as elastic fibers and basement membranes (Zhang et al., 2020). Some researchers have found that EFEMP1 interacts with elastin as well as collagen 15A1 (a component of collagen fibrils) to produce proteins that affect the expression and function of these proteins (Jorgenson et al., 2015). Peng et al. (2022) found reduced EFEMP1 expression in the abdominal fascia of patients with inguinal hernia. Albig et al. (2006) concluded that EFEMP1-deficient female rats had broken elastic fibers in the vaginal wall. The above studies suggest that EFEMP1 has a specific role in the integrity of elastic fibers. The choroid is rich in elastin, and as the choroidal thickness thins due to the growth of the eye axis in myopic patients, the elastin content is altered, which may stimulate the altered expression of EFEMP1. Targeted regulation of EFEMP1 may be useful for investigating the molecular mechanism of CT changes during myopia development.

In summary, OCTA was used to measure the CT thickness of patients with different degrees of myopia, and significant differences were found between groups. Meanwhile, tears of patients with emmetropia and myopia were collected and analyzed, and EFEMP1 concentrations in tears were significantly increased. Subsequently, FDM guinea pigs were used to validate EFEMP1 for CT thickness changes. The expression of EFEMP1 mRNA and protein was upregulated in the choroid of FDM guinea pigs. We speculate that EFEMP1 may be involved in the development of myopia, especially in choroid thickness. EFEMP1 may be a potential target for the prevention and treatment of choroid thickness changes in myopia. However, the limitations of this study are the small sample size collected and the need to further explore the potential mechanisms of EFEMP1 and CT changes in molecular as well as different experimental models in the future.

## Data availability statement

The raw data supporting the conclusions of this article will be made available by the authors, without undue reservation.

## Ethics statement

The studies involving human participants were reviewed and approved by Ethical Committee of Jinshan Hospital of Fudan University (JIEC 2020-S39-02). The patients/participants provided their written informed consent to participate in this study. Written informed consent was obtained from the individual(s) for the publication of any potentially identifiable images or data included in this article.

## Author contributions

W-QS, TW, BL, TL, and X-DZ made substantial contributions to this research. W-QS and BL performed the experiments and collected the data. TW and TL designed the current study. W-QS and X-DZ given final approval of the version to be published. W-QS wrote the manuscript. All authors read and approved the final manuscript.

## Supplementary material

Supplementary Figure 1 ELISA standard curve for the concentration of EFEMP1 in tear fluid.

Supplementary Table 1 Ocular biometric parameters of the right eye measured by A-scan.

Supplementary Table 2 Ocular biometric parameters of the right eye measured by IOL-Master.
