# Supplementary figures and images for: EFEMP1 is a potential biomarker of choroid thickness change in myopia
Source: Front Neurosci. 2023 Feb 20;17:1144421. doi: 10.3389/fnins.2023.1144421 (PMC9987712; doi:10.3389/fnins.2023.1144421)

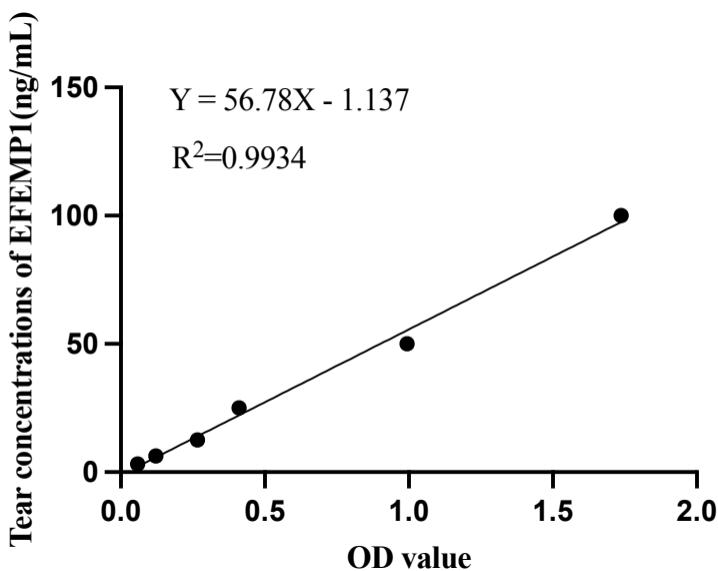

Supplement: Supplementary Figure 1 — ELISA standard curve for the concentration of EFEMP1 in tear fluid. [file Image_1.pdf]
