# Supplementary material for: EFEMP1 is a potential biomarker of choroid thickness change in myopia
Source: Front Neurosci. 2023 Feb 20;17:1144421. doi: 10.3389/fnins.2023.1144421 (PMC9987712; doi:10.3389/fnins.2023.1144421)
Supplement: Supplementary Table 1 — Ocular biometric parameters of the right eye measured by A-scan. [file Table_1.DOCX]

**Table S1** **Ocular biometric parameters of the right eye measured by A-scan.**

| **Parameter** | **EM** | **Non-HM** | ***HM*** | ***P*-value** | ***P*-value_1_** | ***P*-value_2_** | ***P*-value_3_** |
| --- | --- | --- | --- | --- | --- | --- | --- |
| Sample Size | 15 | 46 | 70 | NA | NA | NA | NA |
| AC(mm) | 3.10±0.20 | 3.34±0.33 | 3.40±0.29 | <0.001 | 0.005 | <0.001 | 0.623 |
| LT(mm) | 4.13±0.30 | 3.96±0.32 | 3.91±0.47 | 0.176 | 0.539 | 0.193 | 1.000 |
| VCD(mm) | 16.04±0.50 | 17.23±0.79 | 18.74±1.10 | <0.001 | <0.001 | <0.001 | <0.001 |
| AL(mm) | 23.26±0.32 | 24.52±0.85 | 26.05±1.12 | <0.001 | <0.001 | <0.001 | <0.001 |
| AC/AL | 0.13±0.01 | 0.14±0.01 | 0.13±0.01 | 0.038 | 1.000 | 1.000 | 0.032 |
| VCD/AL | 0.69±0.01 | 0.70±0.01 | 0.72±0.02 | <0.001 | 0.019 | <0.001 | <0.001 |

*P* value among the three groups; *P* Value_1_, *P* value EM and non-HM; *P* Value_2_, *P* value between EM and HM; *P* Value_3_, *P* value between non-HM and HM.EM, emmetropia；HM, high myopia; NA, not applicable；AC, anterior chamber depth;LT, lens thickness;VCD, vitreous chamber depth;AL, axial length.
