# Supplementary material for: EFEMP1 is a potential biomarker of choroid thickness change in myopia
Source: Front Neurosci. 2023 Feb 20;17:1144421. doi: 10.3389/fnins.2023.1144421 (PMC9987712; doi:10.3389/fnins.2023.1144421)
Supplement: Supplementary Table 2 — Ocular biometric parameters of the right eye measured by IOL-Master. [file Table_2.DOCX]

**Table S2 Ocular biometric parameters of the right eye measured by IOL-Master.**

| **Parameter** | **EM** | **Non-HM** | **HM** | ***P*-value** | ***P*-value_1_** | ***P*-value_2_** | ***P*-value_3_** |
| --- | --- | --- | --- | --- | --- | --- | --- |
| Sample Size | 15 | 46 | 70 | NA | NA | NA | NA |
| AL(mm) | 23.46±0.30 | 24.74±0.87 | 26.35±1.12 | <0.001 | <0.001 | <0.001 | <0.001 |
| K1 | 42.84±1.16 | 42.74±2.07 | 43.05±1.75 | 0.670 | 1.000 | 1.000 | 1.000 |
| r1 | 7.88±0.21 | 7.91±0.39 | 7.85±0.32 | 0.630 | 1.000 | 1.000 | 1.000 |
| K2 | 43.62±1.18 | 43.75±2.11 | 44.24±1.90 | 0.295 | 1.000 | 0.767 | 0.546 |
| r2 | 7.74±0.21 | 7.73±0.38 | 7.64±0.33 | 0.300 | 1.000 | 0.891 | 0.499 |
| K | 43.22±1.15 | 43.25±2.07 | 43.64±1.79 | 0.462 | 1.000 | 1.000 | 0.775 |
| CR | 7.81±0.21 | 7.82±0.38 | 7.75±0.32 | 0.449 | 1.000 | 1.000 | 0.694 |
| AL/CR | 3.00±0.06 | 3.17±0.15 | 3.41±0.15 | <0.001 | <0.001 | <0.001 | <0.001 |
| ACD(mm) | 3.42±0.26 | 3.56±0.34 | 3.69±0.29 | 0.003 | 0.402 | 0.007 | 0.071 |
| WTW(mm) | 11.80±0.37 | 11.72±0.42 | 11.79±0.50 | 0.742 | 1.000 | 1.000 | 1.000 |
| ACD/AL | 0.15±0.01 | 0.14±0.01 | 0.14±0.01 | 0.113 | 1.000 | 0.260 | 0.323 |

*P* value among the three groups; *P* Value_1_, *P* value EM and non-HM; *P* Value_2_, *P* value between EM and HM; *P* Value_3_, *P* value between non-HM and HM.EM, emmetropia；HM, high myopia; NA, not applicable；AL, axial length;K, corneal curvature;CR, radius o f corneal curvature;ACD, anterior chamber depth;WTW, white to white.
